# Feasibility of a virtual reality-based interactive feedback program for modifying dysfunctional communication: a preliminary study

**DOI:** 10.1186/s40359-020-00418-0

**Published:** 2020-05-14

**Authors:** Junhyung Kim, Young Hoon Jung, Yu-Bin Shin, Min-Kyeong Kim, Hyojung Eom, Eunjoo Kim, Joohan Kim, Jae-Jin Kim

**Affiliations:** 1 Department of Psychiatry, Yonsei University College of Medicine, Seoul, Republic of Korea; 2 Institute of Behavioral Science in Medicine, Yonsei University College of Medicine, Seoul, Republic of Korea; 3 Department of Communication, Yonsei University College of Social Sciences, Seoul, Republic of Korea

**Keywords:** Dysfunctional communication, Virtual reality, Training program, Feasibility

## Abstract

**Background:**

Functional communication is vital in many areas of daily life, and modifying dysfunctional communication has been emphasized in various social areas, including family and school. The present preliminary study addressed the feasibility of a virtual reality (VR)-based interactive feedback program for the modification of dysfunctional communication.

**Methods:**

Thirty-seven healthy young males completed psychological assessments associated with functional communication and participated in the VR-based program, consisting of the three tasks of ‘exploring the communication style,’ ‘practicing functional communication,’ and ‘expressing empathy.’ Behavioral parameters were recorded based on the participants’ choices among available options and the visual analog scale scores that resulted in responses to questions in the tasks.

**Results:**

Participants completed the program without dropping-out and reported 10.76 (SD, 9.66) in the Simulator Sickness Questionnaire and 106.97 (SD, 16.66) in the Presence Questionnaire. In exploring the communication style, there was no difference between the dysfunction level-with family and dysfunction level-with a friend, but only the dysfunction level-with family showed significant correlations with the level of communication with parents. In practicing functional communication, the communication scores with the placating, blaming, and computing styles significantly increased according to the repetition of trials. In expressing empathy, the empathetic feeling score was negatively correlated with the perspective-taking score, whereas the emotional intensity score was positively correlated with the level of differentiation of the self.

**Conclusion:**

These results suggest that the program may have a tolerable level of cybersickness, an adequate level of presence, an improvement in the behavioral parameters that may reflect the important aspects of communication, and a proper reflection of psychological states or interpersonal characteristics. The use of this program can be an important starting point for the development of a more convenient method for delivering VR programs designed to modify dysfunctional communication, which can further increase computerized dissemination.

## Background

Functional communication is a complex concept defined and used across a variety of domains. Although the definition of functional communication depends on the theorist’s view [1], it is vital in many areas of daily life; receiving crucial information for survival [2], coping with stress through modulating conflicts between one’s desire and environment [3], and maintaining smooth interpersonal relationships [3]. In contrast, dysfunctional communication is known to damage family health [4], mediate increasing problem behaviors [5], and negatively influence partner relationships and further destroy harmony in a family [6]. Therefore, modifying dysfunctional communication has been emphasized in various social areas, including family [7], couples [8], health care workers [9], and schools [10]. Previous studies have suggested that several psychological factors, such as empathy, differentiation of self, and parent-adolescent communication, should be considered in the modification of dysfunctional communication [11–13].

Virginia Satir, one of the well-known theorists of functional communication, investigated the communication type and suggested the mechanism of change for such types of communication [14]. In Satir’s model, typologies of dysfunctional communication are clearly defined; placating, blaming, computing, and distracting [15, 16]. Placaters are non-assertive, avoid conflict, always seek approval, and are sensitive to how others perceive them. Blamers are self-assertive without taking account the others’ position and always blame someone or something else. Computers use intelligence to analyze, plan, and solve problems, and thus appear cold or unfeeling. Distracters seek attention to compensate for their feelings of inadequacy and use a range of negative emotions to either avoid an issue or manipulate how others feel. Although this model primarily focused on family therapy, it has been expanded into the more general area of communication because of its empirical, solution-focused, and spiritual nature [17]. According to the model, clear factors which prevent individuals from fully delivering their thoughts and emotions are associated with a difference between functional and dysfunctional communications [18, 19] and dysfunctional communication can be modified by investigating the feelings, perceptions, and cognitive expectations of self in the here-and-now experience [20–22]. Various studies have shown that the modification of dysfunctional communication was effectively employed using this model in learners-oriented education methods or role-playing for improving communication skills [23, 24].

Despite the benefits of Satir’s theory in modifying dysfunctional communication, individual or group interventions offered at schools or workplaces are not always accessible (e.g., rural communities or small businesses) or sustainable (e.g., cost concerns). Virtual reality (VR) may be the adequate technology to be used conveniently anytime and anywhere for a positive intervention for modifying dysfunctional communication. VR can provide a realistic environment for individuals to communicate with others and it has a great potential to objectively measure individuals’ cognitive or emotional behaviors in everyday life [25–28]. VR has already been utilized in the treatment of various psychiatric or medical conditions, such as anxiety disorder [29], depression [30, 31], pain [32, 33], and traumatic brain injury [34]. In addition, behavioral therapy in virtual environments for improving communication skills has been utilized for patients with some psychiatric disorders: emotional/social adaptation skills training for those with autism spectrum disorder [35] or schizophrenia [36] and presentation skills training for those with social anxiety disorder [37].

The clinical application of VR in interventions, based on psychological theory associated with delivering educational content and the correction of cognitive distortion, is effective in improving the symptoms of depression and anxiety disorders [30]. Interventions using VR are accessible to be applied in experiential learning for various education systems [38, 39]. Delivering educational content based on a practical and valid communication theory using VR may also be effective in modifying dysfunctional communication in the general population. Nonetheless, a VR intervention for the modification of dysfunctional communication has not yet been reported. Satir’s model may be appropriate in this intervention due to its characteristics of emphasizing the here-and-now experience in interventions [35, 37]. Based on this background, we developed a VR training program targeted for modifying dysfunctional communication in the general population.

Considering the complexity of communication and the need to explore the applicability of VR, the current study addressed the feasibility of the VR training program for the modification of dysfunctional communication. The purposes of this pilot study were to explore whether this training program could be carried out, if it would show any evidence of efficacy in modifying dysfunctional communication, and if the responses of participants in the training program could reflect their psychological characteristics associated with functional communication. For these purposes, we examined the level of acceptance and perceived utility, as well as the possible benefits of this training program. The hypotheses were that 1) most participants would accept this program without any drop-out, with tolerable cybersickness, and an acceptable level of presence in VR, 2) participants would show evidence suggesting improvements in behavioral parameters from the program, and 3) the behavioral parameters from the program would demonstrate significant relationships with psychological assessments related to functional communication.

## Methods

### Participants

Participants were 37 healthy adult volunteers, who were recruited through on-line advertisements on a university announcement board. We confined participants to unmarried, young males, only to focus on acceptance and perceived utility of our VR interactive feedback program without considering an issue of gender or culture in interpersonal communication [40]. Additional exclusion criteria were the current use of psychotropic medications and any history of substance use disorder, neurological or neurodevelopmental disorder, major depressive episodes, bipolar I disorder, or psychotic disorders. The application of these criteria was achieved through an interview with a psychiatrist using the Mini-International Neuropsychiatric Interview [41]. The majority of participants were university students (*N* = 35) and the mean age of participants was 22.20 (SD, 13.20). The average level of education was 14 years (SD, 1.31). All participants gave written informed consent after being informed about the procedure of the study. This study was approved by the Institutional Review Board of Yonsei University Gangnam Severance Hospital, South Korea.

### Study procedures

**Fig. 1:**
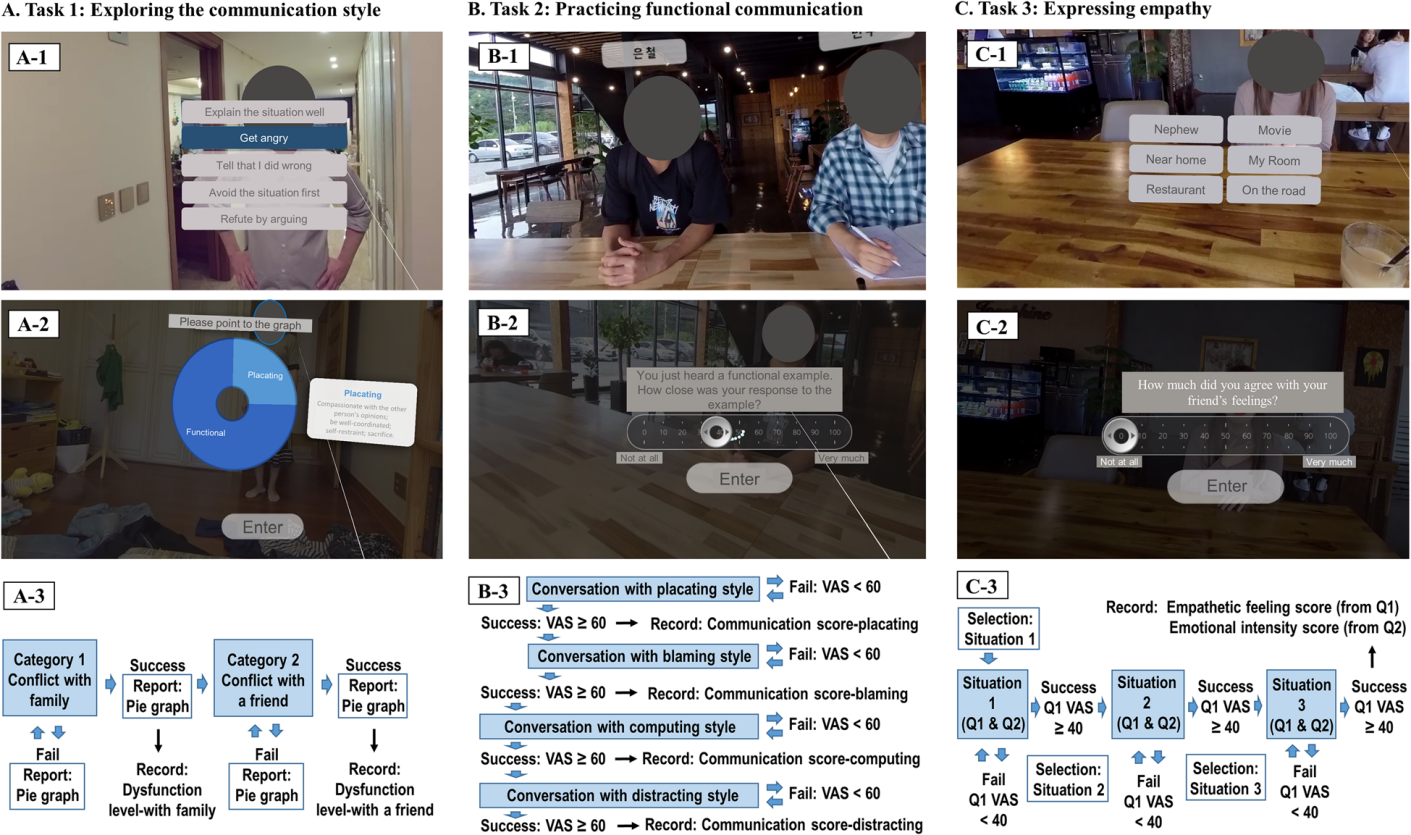
Screenshots and schematic diagrams of the virtual reality-based interactive feedback program, “Enhancing Functional Communication,” that includes three different tasks. The program was conducted in Korean, but the examples of the help balloon in the figure are presented in English to aid readers understanding. To protect the portrait rights of the cast, the face is obscured. VAS, visual analog scale; Q, question

All participants experienced the VR-based interactive feedback program of about 45 min, ‘Enhancing Functional Communication’ (Fig. 1) once. The program ran on a VR system consisting of a desktop computer containing an NVIDIA GeForce GTX 970 graphics card and 16 GB RAM of graphics memory, running the Microsoft Windows 10 operating system, and equipped with an Oculus Rift head-mounted display with a tracker (Oculus VR, LLC, USA). The Touch Controller (Oculus VR, LLC, USA), a new device suitable for hand gesture-controlled user interfaces, was used for interactions with executable objects and avatars during the virtual experience. All tasks in the program were produced using a video shot of the acting of professional actors according to a pre-made scenario, which was filmed at a resolution of 7680 × 3840 with a 360-degree camera (Insta360 Pro, Insta360, China). Autopano (Kolor, USA) and Adobe Premiere Pro (Adobe Inc., USA) were used to edit the recorded video at a resolution of 4096 × 2048 and insert the built-in voice or text guidance.

The program consisted of three tasks: ‘Exploring the communication style,’ ‘Practicing functional communication,’ and ‘Expressing empathy.’ The scenarios for the situations in each task are provided in Table 1. In all tasks, participants were advised to consider and talk in detail as they deemed appropriate in a given interpersonal situation. Participants performed the tasks while sitting in a chair in a quiet room. An assistant provided only help with the operation of VR equipment.

**Table 1:** Summary of situations presented in the three tasks of the virtual reality-based interactive feedback program, ‘Enhancing Functional Communication’

Situation	Content
*1. Exploring the communication style*	
Conflict with father	The virtual situation assumed that the participant could not contact his/her parents because the cell phone was turned off while studying for an exam in the library. Therefore, his/her father was angry because he/she was out of reach until late at night. The participant talked with the virtual father at the front gate after he/she got home.
Conflict with mother	The virtual situation assumed that the participant ignored his/her mother’s request for cleaning up his/her room and the mother was angry due to his/her clear fault. The participant talked with the virtual mother, who had just seen his/her messy room.
Conflict with a friend who misunderstood	The virtual environment was based on a hypothetical situation, where a virtual close friend, “Jie-Hye”, misunderstood the participant as having talked behind Jie-Hye’s back with another friend even though he/she did not do this. The participant talked with Ji-Hye about the misunderstanding in a café.
Conflict with a friend who was late for an appointment	The virtual situation assumed that the participant was waiting for a virtual friend, “Jung-Woo”, who was late for an appointment due to a valid reason; and another virtual friend, “Young-Soo”, was angry at Jung-Woo for being late. The participant talked with Jung-Woo, who just arrived at a café.
*2. Practicing functional communication*	
with a placating style	The virtual situation assumed that the participant discussed the ideas of presentation for school classes with their group as a facilitator. Before the virtual situations, the participant was informed that one of the group members had a placating communication type, named Soo-Jie. Beginning of the simulation, Soo-Jie was seen hesitant to express her opinions, and the other members of the group were clamoring for her opinion. The mission began when another group member asked the participant to encourage Soo-Jie to express her opinion.
with a blaming style	The virtual situation assumed that a friend with a blaming communication type named Jeong-Woo, who is not very close to the participant, asked another friend to show homework while the participant was doing one’s own homework at school. During the virtual situation, Jeong-Woo did not explain why he couldn’t do his work. After another friend refused his request, he swore and showed a sign of aggression toward the friend. The mission began when Jeong-Woo asked the participant to show the participant’s homework.
with a computing style	The virtual situation began with listening to a disagreeable experience of a friend with a computing communication type, named So-Jeong. Because So-Jeong had occupied two seats with her cello on a crowded train on the weekend after her cello concert, she had been scolded by a strange older man. There was no legal problem with her standard purchase of tickets. However, the conflict might have grown because she only had focused on the situation without understanding the other’s feelings and did not communicate her circumstances and emotions appropriately. The mission began when So-Jeong asked the participants to share their thoughts about her experience.
with a distracting style	The virtual situation assumed that the participant had plans to study for a test with a couple of friends, Joon-Young and Jie-Young, but they were late for the appointment. They were fighting ever since they had arrived. Joon-Young, who has a distracting communication type, was late without a good excuse, but only tried to avoid the situation by ignoring Jie-Young’s continued questions. The mission began when Jie-Young asked the participant to persuade Joon-Young to confront the situation appropriately.
*3. Expressing empathy*	
Nephew	A friend recounts his first experience of being called as an uncle by his nephew.
Near home	A friend shares the terrifying experience of a suspicious person chasing after her on her way home last night.
Restaurant	A friend recounts the surprising experience when his favorite celebrity was seated next to him at a restaurant the day before.
Movie	A friend recounts the sadness she felt in a movie when the loving couple had to break up due to illness.
My room	A friend recounts the disgusting experience of seeing a small but many-legged bug and getting goose bumps.
On the road	A friend complains angrily about a man who bumped into him and scolded him on the street the day before.

#### The task of exploring the communication style

**Fig. 2:**
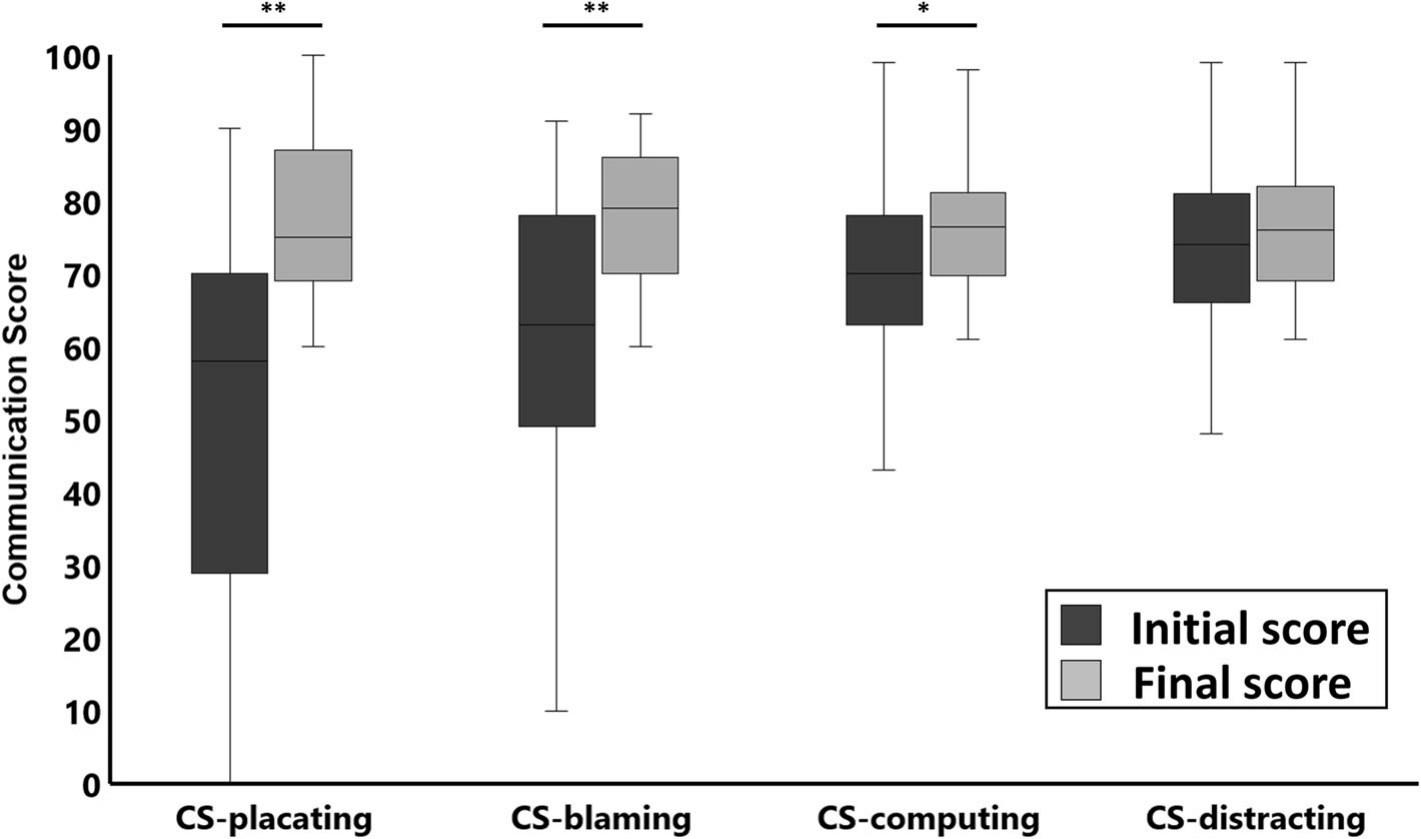
Changes in the behavioral parameters at the initial and final trials in the task of practicing functional communication. Standard errors are represented as error bars. *p < 0.05, **p < 0.001

**Fig. 3:**
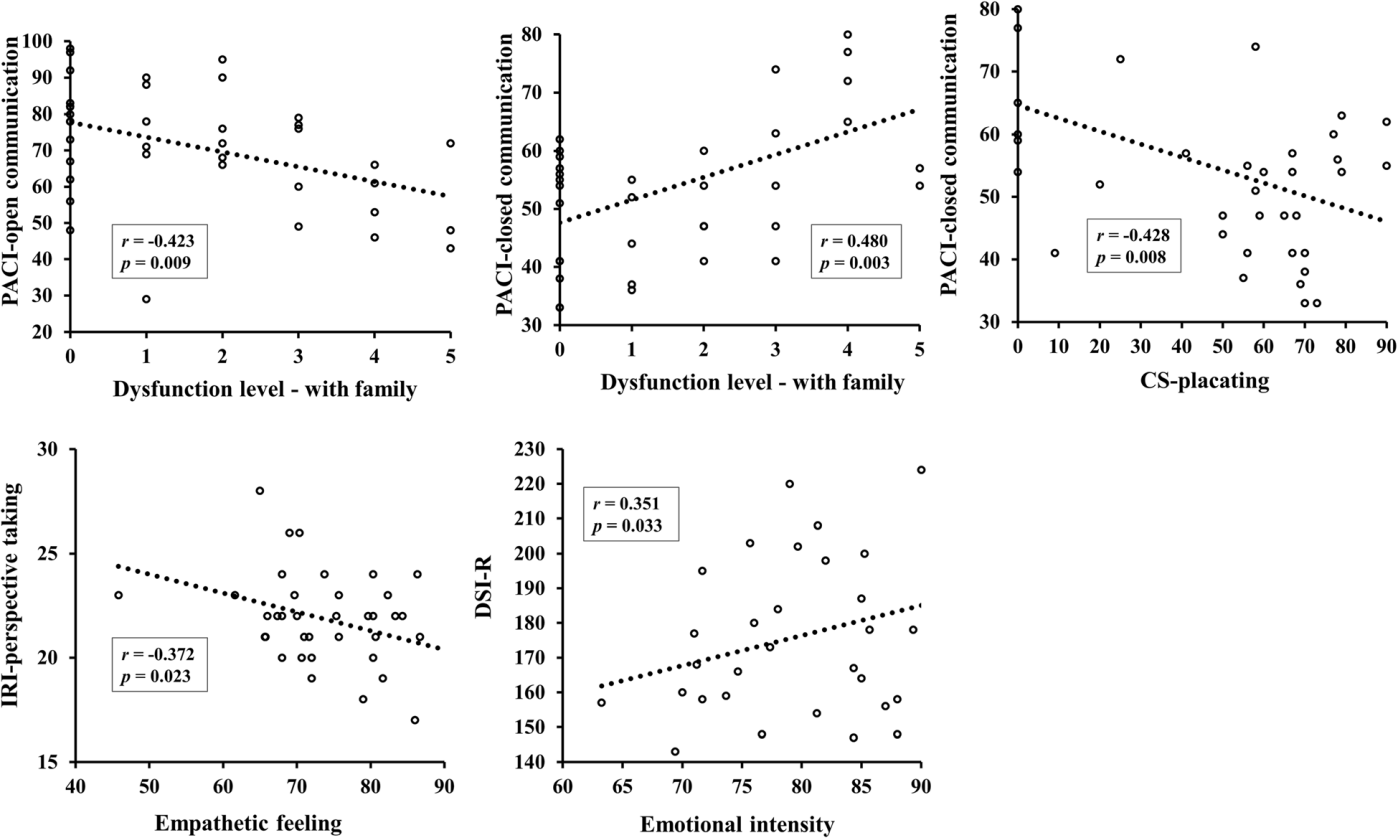
Significant correlations between the behavior parameters in the virtual reality-based interactive feedback program and the psychological assessment scale scores related to communication and interpersonal relationship. PACI, Parent Adolescence Communication Inventory; CS-placating, communication score with a placating style

The purpose of this task was to allow participants to evaluate their communication style in an interpersonal situation and to practice the functional communication style with voice and text guidance. The task consisted of two categories (‘Conflict with family’ and ‘Conflict with a friend’), each containing two conflict situations (with the father and mother and with a female friend and male friend, respectively). After the voice guidance explanation of the conflict, a virtual person (one of the parents or friends) spoke emotional words to participants. They had to choose from one of five options that reflected the style of communication based on Satir’s theory (placating, blaming, computing, distracting, and functional) ((Fig. 1_A-_1), and the corresponding recorded words were presented as their response and the virtual person’s recorded words corresponding to their choice were followed. Then, the next situation with another virtual person in the category was presented and proceeded in the same way. If participants chose the option of functional communication in both situations, they could finish the category. If they chose a dysfunctional communication style in any situation, participants had to repeat the two situations until they choose only the option of functional communication. At every end of a category, a pie graph was presented as a report of the communication style (Fig. 1_A-_2) and advice for functional communication was provided when participants clicked on the graph. The numbers of dysfunctional communication-related choices made before the completion of the category were recorded and referred to as the ‘dysfunction level-with family’ and ‘dysfunction level-with a friend,’ respectively (Fig. 1_A-3_).

#### The task of practicing functional communication

In this task, participants learn a way to functionally communicate with someone who has a dysfunctional communication style in four different conflict-driven situations. When participants started the task, they were positioned in a virtual study-room. In each conflict situation, voice guidance explained a state that participants were asked to think and talk about, and the environment was changed to a scene interacting with two or three others (Fig. 1_B-1_). One of them spoke to participants in one of four dysfunctional communication styles (placating, blaming, computing, or distracting), and participants responded freely to him/her. Then, the voice guidance explained a problem of presented dysfunctional communication and a possible response of functional communication was played as an example. Evaluation after completing the task was based on a question: “How close was your response to the example?” Participants’ responses were rated on a visual analog scale (VAS), which presented ‘not at all’ (0 points) at the left end of a horizontal line and ‘very much’ (100 points) at the right end (Fig. 1_B-2_). The VAS score was referred to as the communication score with the placating, blaming, computing, or distracting style (abbreviation: CS-placating, CS-blaming, CS-computing, and CS-distracting, respectively). If the communication score was less than 60, participants were asked to re-try the situation until they scored 60 or more (Fig. 1_B-3_). The score on the first attempt in each situation was recorded as the initial score. If the task was performed more than once, the score of the last performance was recorded as the final score and the number of trials was also recorded.

#### The task of expressing empathy

This task was developed to enhance functional communication by trying to understand the other’s feelings and express empathic concerns. In the beginning, participants were placed in a virtual café with a friend. If participants chose one of six options, such as Nephew, Near home, Restaurant, Movie, My room, and On the road (Fig. 1_C-1_), the friend told them about an event that caused a feeling of pleasure, fear, surprise, sadness, disgust, and anger, respectively. Participants were asked to select and perform three situations among the options. The reason for selecting only three situations was to prevent participants from being distracted due to excessive experiment time, considering that the current study was a preliminary feasibility test. During each situation, participants should listen to his/her story carefully, grasp his/her feelings, and say what they wanted to tell him/her. Then, participants were prompted to score the VAS scale in response to two questions: “How much did you agree with your friend’s feelings?” (Fig. 1_C-2_) and “How strong was your friend’s emotion?” In each situation, if the score for the first question was less than 40, participants were asked to repeat the situation until they scored 40 or more. The number of trials was recorded. Despite the repetition, only the VAS score from the initial trial of the three performed situations was used in the analysis and the mean of the three VAS scores was referred to as the empathetic feeling score and emotional intensity score for the two respective questions (Fig. 1_C-3_).

### Psychological assessments

Three different self-report scales were used for evaluating the participants’ psychological states. The Parent Adolescence Communication Inventory (PACI), with a 20-item 5-point Likert scale, was used to assess the level of communication with parents on the open or closed dimension [42]. The higher open score corresponds to more freely expressing the thoughts and feelings without being oppressed, whereas the higher closed score represents more hesitance to express the opinions and more careful selection of dialogue material. In order to measure the level of empathy, we used the Interpersonal Reaction Index (IRI), with a 28-item 5-point Likert scale, that contains four subscales (fantasy, perspective-taking, empathetic concern, and personal distress) with seven items for each subscale [43]. In the current study, only two subscales, including perspective-taking and empathetic concern, were used to examine the tendency to adopt the other’s psychological viewpoint (the cognitive aspect of empathy) and the feelings of compassion and sympathy for others (the emotional aspect of empathy), respectively [44]. We also used the Differentiation of Self Inventory-Revised (DSI-R), with a 46-item 6-point Likert scale [45], in which high scores indicate a high level of differentiation of the self.

Two different self-report scales were used for investigating the usability of VR. The Simulator Sickness Questionnaire (SSQ), a 16-item questionnaire, was used to examine the occurrence and severity of cybersickness symptoms when immersed in virtual environments [46]. The weighted mean SSQ score of the projection type of the head-mounted display used in the current study was rated as 29.9 [47]. The Presence Questionnaire (PQ), a 29-item questionnaire, was used to measure the presence level of the VR experience [48]. The score of this questionnaire ranges from 29 to 203, and can be graded as 0–67 as low, 68–133 as medium, and ≥ 134 as high [49, 50]. Considering the nature of our program, only 22 items related to involvement, adaptation/immersion, or interface quality were included in the analysis, excluding the sensory fidelity, and thus the range of this modified version was 22–154.

### Statistical analysis

Descriptive statistics were used to explore the demographic characteristics, psychological characteristics related to communication, the sense of cybersickness and presence in the VR experience, and the parameters from the number of trials obtained during the performance of each of the three tasks. Paired t-tests were used to compare the dysfunction level-with family and dysfunction level-with a friend in the task of exploring the communication style and to compare the initial and final scores in the task of practicing functional communication for investigating the possible benefits of the program. Pearson correlation coefficients between behavior parameters and psychological assessments were calculated for supporting the possible benefits and investigating the concurrent validity of the behavior parameters. Statistical significance was accepted at an alpha level of 0.05. All data were analyzed using the Statistical Package for the Social Sciences 25.0 (SPSS Version 25.0; IBM Corporation, Armonk, NY, USA).

## Results

### Descriptive statistics of the psychological scale scores and number of task trials

The descriptive statistics for all psychological scale scores obtained in the study are shown in Table 2. All of the participants completed all tasks in the program without giving up. The mean SSQ and PQ scores were 10.76 (SD, 9.66) and 106.97 (SD, 16.66), respectively.

**Table 2:** Descriptive statistics of psychological assessments (*n* = 37)

Psychological assessments	Mean	Standard deviation
Parent Adolescence Communication Inventory		
Open communication	70.70	16.43
Closed communication	54.38	13.79
Interpersonal Reaction Index		
Perspective-taking	21.81	2.27
Empathic concern	23.81	2.53
Differentiation of Self Inventory-Revised	178.79	21.89
Simulator Sickness Questionnaire	10.76	9.66
Presence Questionnaire	106.97	16.66

The mean number of trials in the task of practicing functional communication was 1.54 (SD, 0.51) in communication with placating, 1.43 (SD, 0.55) in communication with blaming, 1.19 (SD, 0.40) in communication with computing, and 1.11 (SD, 0.31) in communication with distracting. The mean number of trials in the task of expressing empathy was 1.08 (SD, 0.27).

### Possible benefits of the program

In the task of exploring the communication style, no significant difference was found between the dysfunction level-with family and dysfunction level-with a friend [1.73 (SD, 1.69) and 1.68 (SD, 1.45), *t* = 0.161, *p* = 0.873]. In the task of practicing functional communication, the mean CS-placating was initially 49.65 (SD, 28.56) and significantly increased to 77.43 (SD, 10.85) at the final performance (*t* = 5.62, *p* < 0.001). The mean CS-blaming and CS-computing also significantly increased at the final performance [59.84 (SD, 23.99) and 78.00 (SD, 9.28), *t* = 4.56, *p* < 0.001; 70.47 (SD, 11.74) and 76.47 (SD, 9.87), *t* = 2.51, *p* = 0.017, respectively], whereas the mean CS-distracting did not significantly increase [73.76 (SD, 11.80) and 75.78 (SD, 9.85), *t* = 1.68, *p* = 0.102] (Fig. 2).

### Relationship between the behavior parameters and psychological assessments

The correlations between the parameters from the task and psychological assessments are presented in Table 3. In the task of exploring the communication style, the dysfunction level-with family was significantly correlated with both dimensions of the PACI (open communication: *r* = − 0.423, *p* = 0.009; and closed communication: *r* = 0.480, *p* = 0.003), but there was no significance in the correlations with the dysfunction level-with a friend. The only significant correlation of the parameters from the task of practicing functional communication was found between the CS-placating and the PACI-closed communication score (*r* = − 0.428, *p* = 0.008). Significant correlations of the parameters from the task of expressing empathy were identified only in the two pairs: between the empathetic feeling score and IRI-perspective-taking score (*r* = − 0.372, *p* = 0.023) and between the emotional intensity score and the DSI-R score (*r* = 0.351, *p* = 0.033) (Fig. 3). Meanwhile, there was no significant correlation between the task parameters and the PQ or SSQ score.

**Table 3:** Correlations between the behavior parameters and psychological assessments (*n* = 37)

	Dysfunction level	CS-placating	CS-blaming	CS-computing	CS-distracting	Empathetic feeling	Emotional intensity
PACI							
Open communication	−0.23	0.265	0.252	−0.015	−0.062	0.046	0.029
Closed communication	0.337*	−0.428*	− 0.085	0.105	0.061	0.001	−0.062
IRI							
Perspective taking	0.085	−0.034	−0.131	0.023	−0.162	− 0.372*	−0.0155
Empathic concern	−0.229	−0.024	0.003	0.091	−0.021	−0.275	− 0.004
DSI-R	−0.158	0.029	0.121	−0.125	0.079	0.101	0.351*
Presence scale	0.230	0.254	0.025	0.063	0.082	0.259	0.074
SSQ	0.230	−0.294	0.040	0.246	0.073	0.215	0.147

*CS* communication score, *PACI* Parent Adolescence Communication Inventory, *IRI* Interpersonal Reaction Index, *DSI-R* Differentiation of Self Inventory-Revised, *SSQ* Simulator Sickness Questionnaire. * *p* < 0.05

## Discussion

The current preliminary study explored the feasibility of a VR program for the modification of dysfunctional communication in young adults. In the respect of acceptability, our sample of 37 non-clinical participants attending the current study completed the program without giving up. The mean SSQ score of 10.7 was very low compared to the previously reported average score of 29.9 [47], suggesting that our program causes an acceptable level of cybersickness. Although 7 items were excluded from the original questionnaire in the current study, the mean of scores was 106.97 (SD, 16.66) that was rated as the medium level of the original PQ. Given that the presence in VR is an important factor in VR-based trainings [51, 52], this result may support the usability of our program. Although the direct positive impact of this presence on training cannot be determined due to the absence of significant correlations between the PQ score and task parameters, the experience of VR based on the presence may maximize the usefulness of this program by combining with other advantages of VR, such as surpassing spatial limitations and providing interactive feedback information.

For completing each category of ‘Conflict with family’ and ‘Conflict with a friend’ in the task of exploring the communication style, participants had to repeat the task until they choose a functional communication style in all situations of each category. As a result, the more options of dysfunctional communication were chosen, the more advices were given on finding the functional communication style. We designed this program in a way that provided this unequal information because it was created for training people with high dysfunction level. Given that there is a difference in individuals’ interpersonal behaviors between family and friends [53], we assumed that there might be a difference between the dysfunction level-with family and dysfunction level-with a friend. However, there was no significant difference between the two types of dysfunction level, suggesting that this task was not sensitive enough to detect such differences in interpersonal behaviors. Nevertheless, only the dysfunction level-with family showed significant correlations with the open and closed dimensions of the PACI. These results suggest the possibility of using our VR task to objectively measure how family members communicate.

In order to complete the task of practicing functional communication and the task of expressing empathy, participants had to self-evaluate their performance in each situation to be over the preset score. Because participants had to listen to examples and guidance related to functional communication in each iteration for training the modification of dysfunctional communication, the number of trials could be interpreted as reflecting their effort to accept the content of the program. The number of trials was no more than three in all situations in two tasks, suggesting that most participants satisfyingly accepted the content of the program and tried to functionally communicate with someone. As in other training examples [54, 55], how many times the task was completed could be an important indicator of training. Since participants in the current study were healthy volunteers without psychiatric illness, they seem to be able to complete the tasks with little effort. If a large number of people with interpersonal communication problems participated in the program, the number of trials is likely to increase even further.

For investigating the possible benefits of the program, we compared the initial and final communication scores with the dysfunctional communication styles in the task of practicing functional communication. The communication scores significantly increased with the repetition of the task, except for the CS-distracting. This score reflects how much participants felt able to communicate functionally with someone with a dysfunctional communication style, and thus it is assumed that participants could realize a way to functionally communicate with individuals showing different communication styles through the repetition of tasks. In particular, the CS-placating showed negative correlations with closedness of communication with parents measured by the PACI-closed communication subscale. Because individuals with lower PACI closedness scores may more easily express their feelings and thought and tend to be more cooperative interpersonal relationships [56], the CS-placating might reflect the tendency of less closedness and more cooperative interpersonal relationships. Therefore, our result may support the potential benefits of practicing functional communication in modifying dysfunctional communication, particularly with the placating communication style.

The CS-distracting probably showed no change because the baseline score was already high. This result may reflect that participants felt relatively comfortable with communicating functionally with the distracting communication style. With regard to this interpretation, one thing to consider is that, unlike the other situations, communication with the distracting communication style included the problem that occurred regardless of the participant. Because individuals tend to be more sensitive to directly self-related situations due to the self-referencing effect [57], it can be inferred that participants might feel more comfortable when communicating the problem that occurred regardless of themselves.

The mean number of trials in the task of expressing empathy was very low at 1.08 because only four participants performed one additional trial to complete the task. Most participants seemed to feel that they could be empathetic with the words of others under the proposed virtual situations in the task. In the correlation analysis, the empathetic feeling score showed a negative correlation with the IRI-perspective-taking score, whereas the emotional intensity score showed a positive correlation with the DSI-R score. The perspective-taking domain of the IRI and DSI-R evaluate the cognitive parts of an individual’s ability to empathize [44] and the level of self-differentiation [45], respectively. Because both being empathetic and good self-differentiation are vital in functional communication [12, 13], the behavior parameters in the task of expressing empathy may reflect the important aspects of functional communication. These parameters can, therefore, be used to monitor the changing level in the training for improving empathetic ability.

In all of the three tasks, participants had to repeat the trials until they met a predetermined criterion. Thus, the tasks implied demand characteristics that induced participants’ response in the direction of the correct answer. The adoption of this method was because the purpose of our program was not to be used as an evaluation tool, but as a therapeutic training to help people with dysfunctional communication to experience functional communication. Since our participants were healthy young adults, they realized these demand characteristics relatively easily. However, if participants were patients with a serious communication problem, such realization may be more difficult. This will be the subject of our future research.

Although the findings are encouraging, the current study has several limitations. Our VR program introduced a feedback method to improve functional communication between people, but the interaction function of the way people communicate with each other was not equipped. This could only be possible in future advanced versions with the help of artificial intelligence technology. The current study was based on a one-time experience of the VR program rather than a repetitive exercise, and thus changes in communication skills before and after the experience were not measured. Additionally, this short experience is not enough for the program to be truly used for training purposes. Scheduling for repeated use and additional training materials may be required. Because acceptability/feasibility studies cannot provide a scientifically conclusive interpretation by nature [58], the results should be interpreted with caution. The small sample size, only including young healthy males, the within-subject experimental design, and the absence of a control group also limit the generalization of results. To be used for training purposes, a more intensive applicability study should be conducted with a more diverse sample; including a broad range of mental disorders, ages, and females. In addition, a systematic repeating schedule and objective evaluation systems using biosignals are also needed.

## Conclusion

This study provides evidence that the VR-based interactive program for modifying dysfunctional communication has acceptability/feasibility; a tolerable level of cybersickness, an adequate level of presence in VR, with the improvement of behavior parameters which may reflect the important aspects of communication, and the feasibility of evaluation in the program. Obtaining such evidence is a step forward in applying Satir’s theory to individuals suffering from dysfunctional communication. Meanwhile, since the manner in which situations are directly related to oneself can affect the participant’s response, it is necessary to ensure that situations of the scenarios are consistent in the future to improve the reliability of the assessment. We believe that the use of our program will be an important starting point for the development of more convenient methods for delivering VR programs for modifying dysfunctional communication, which can increase computerized dissemination.

## Data Availability

All data generated or analyzed during this study are included in this published article. Further material details are available from the corresponding author on reasonable request.

## Abbreviations

CS: Communication score
DSI-R: Differentiation of Self Inventory-Revised
IRI: Interpersonal Reaction Index
PACI: Parent Adolescence Communication Inventory
PQ: Presence Questionnaire
SSQ: Simulator Sickness Questionnaire
VAS: Visual analog scale; VR: virtual reality
